# Retrospective Study of Metastatic Melanoma and Renal Cell Carcinoma to the Brain with Multivariate Analysis of Prognostic Pre-Treatment Clinical Factors

**DOI:** 10.3390/ijms17030400

**Published:** 2016-03-18

**Authors:** Ethan A. Ferrel, Andrew T. Roehrig, Erin A. Kaya, Jonathan D. Carlson, Benjamin C. Ling, Aaron Wagner, Alexander R. MacKay, Jason A. Call, John J. Demakas, Wayne T. Lamoreaux, Robert K. Fairbanks, Barton S. Cooke, Ben Peressini, Christopher M. Lee

**Affiliations:** 1 Gamma Knife of Spokane, 910 W 5th Ave, Suite 102, Spokane, WA 99204, USA; ferrele@uw.edu (E.A.F.); roehriat@uw.edu (A.T.R.); erinkaya@berkeley.edu (E.A.K.); jonathan.carlson@providence.org (J.D.C.); aaron.wagner@ccnw.net (A.W.); gkspokane@armackay.net (A.R.M.); jdemakas@rockwoodclinic.com (J.J.D.); wayne.lamoreaux@ccnw.net (W.T.L.); fairbrk@ccnw.net (R.K.F.); bo@gkspokane.com (B.S.C.); 2 Cancer Care Northwest, 910 W 5th Ave, Suite 102, Spokane, WA 99204, USA; jason.call@ccnw.net; 3 University of Washington School of Medicine, 1959 NE Pacific St, Seattle, WA 98195, USA; 4 Inland Neurosurgery & Spine Associates, 105 W 8th Ave, Suite 200, Spokane, WA 99204, USA; benjamin.ling@providence.org; 5 Rockwood Clinic, 801 W. 5th Ave, Suite 525, Spokane, WA 99204, USA; 6 DataWorks Northwest, LLC, 3952 N Magnuson St, Coeur D’Alene, ID 83815, USA; benperessini@dataworksnw.com

**Keywords:** melanoma, renal cell carcinoma, brain metastasis, stereotactic radiosurgery, prognostic factors

## Abstract

Patients with brain metastasis from renal cell carcinoma (RCC) or melanoma have historically had very poor prognoses of less than one year. Stereotactic radiosurgery (SRS) can be an effective treatment for patients with these tumors. This study analyzes the effect of pretreatment prognostic factors on overall survival (OS) for RCC and melanoma patients with metastasis to the brain treated with SRS. A total of 122 patients with brain metastases from either RCC or melanoma were grouped by age at brain metastasis diagnosis, whether they received whole brain radiation therapy (WBRT) in addition to SRS, or they underwent surgical resection, Karnofsky Performance Score (KPS), number of brain metastases, and primary tumor. Median survival times for melanoma patients and RCC patients were 8.20 ± 3.06 and 12.70 ± 2.63 months, respectively. Patients with >5 metastases had a significantly shorter median survival time (6.60 ± 2.45 months) than the reference group (1 metastasis, 10.70 ± 13.40 months, *p* = 0.024). Patients with KPS ≤ 60 experienced significantly shorter survival than the reference group (KPS = 90–100), with median survival times of 5.80 ± 2.46 months (*p* < 0.001) and 45.20 ± 43.52 months, respectively. We found a median overall survival time of 12.7 and 8.2 months for RCC and melanoma, respectively. Our study determined that a higher number of brain metastases (>5) and lower KPS were statistically significant predictors of a lower OS prognosis.

## 1. Introduction

Secondary metastasis to the brain continues to be a common cause of death in cancer patients. An estimated 20%–40% of newly diagnosed cancer patients develop brain metastases annually [1,2,3,4]. Brain metastases are malignant tumors which originate from primary cancer elsewhere in the body and can cause severe neurologic compromise, eventually leading to death [5]. Increasingly aggressive therapies targeting systemic disease and advances in diagnostic imaging have led to more frequent and early diagnoses of metastatic tumors [1,2,3,4].

Melanoma and renal cell carcinomas commonly metastasize to the central nervous system, with melanoma as the third most common source of brain metastasis [6]. Each year in the U.S., roughly 170,000 new cases of intracranial metastasis are diagnosed, an estimated 1200–5100 of which are in renal cell carcinoma patients and 17,000 in patients with melanoma [1,2,3,4,7]. Standard treatments for these lesions includes corticosteroids, surgery, whole brain radiotherapy (WBRT), and stereotactic radiosurgery (SRS) [8]. Intracranial metastases from renal cell carcinoma and melanoma seem to respond poorly to WBRT, possibly due to these cell types being more radio-resistant tumor histologies [9,10]. In comparison, SRS has proven to be an effective addition to standard treatments, and even treatment with SRS alone has shown to result in comparable local control and survival outcomes when compared with WBRT or combination regimens [11,12,13].

Still, most patients with these lesions have a poor prognosis. The average survival time for melanoma patients with untreated brain metastasis is less than one month and two to eight months if treated. Renal cell carcinoma patients are expected to survive three months untreated and two to nine months if treated with WBRT [6,14,15,16,17,18,19]. The optimal treatments for these patients remains the subject of much ongoing research [20]. Maximizing overall survival (OS), neurologic capacity, and general comfort is of great interest to clinicians.

Properly selecting patients for appropriate treatment modalities requires an analysis of various prognostic clinical factors. Numerous studies have identified correlations between pre-treatment factors and survival times, but few have attempted to relate survival times to pre-treatment factors specifically in renal cell carcinoma (RCC) or melanoma patients undergoing SRS [21,22,23]. Given the documented benefit of SRS in treatment of these metastases, it is necessary to examine prognostic factors in patients undergoing SRS to understand how to most effectively treat future patients.

We offer an analysis of 122 patients with intracranial RCC or melanoma treated with Gamma Knife radiosurgery (GKRS) alone or GKRS + WBRT at Gamma Knife of Spokane. The purpose of this study is to understand the impacts of several pre-treatment clinical factors on prognosis in patients undergoing these treatments.

## 2. Results

Demographic data of the 122 examined patients are shown in Table 1.

**Figure 1:**
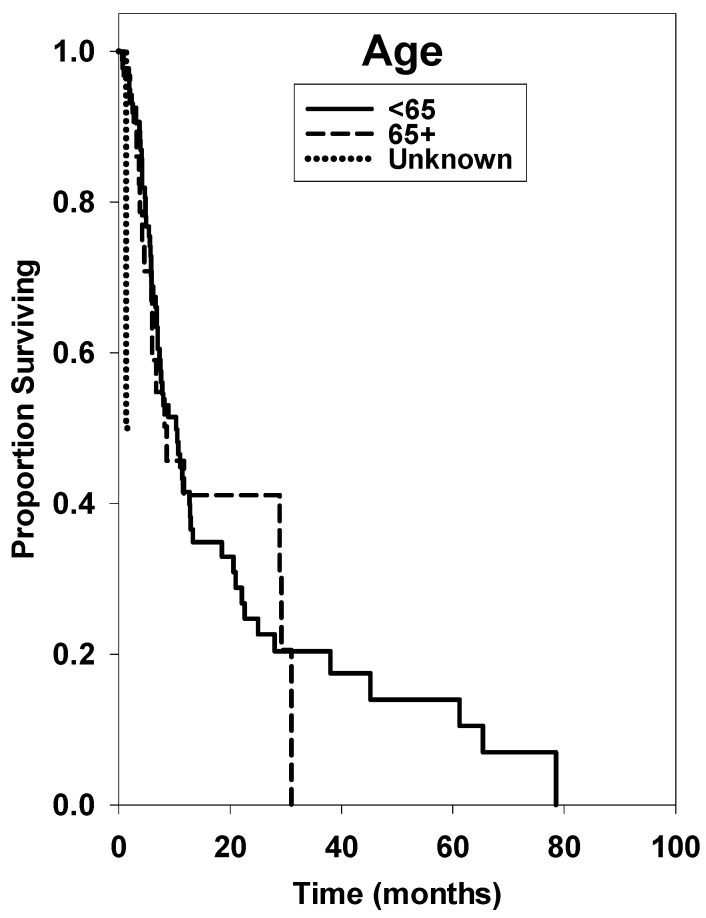
Overall survival (OS) curves of patients with ages <65, 65+, or unknown at time of brain metastasis diagnosis.

**Figure 2:**
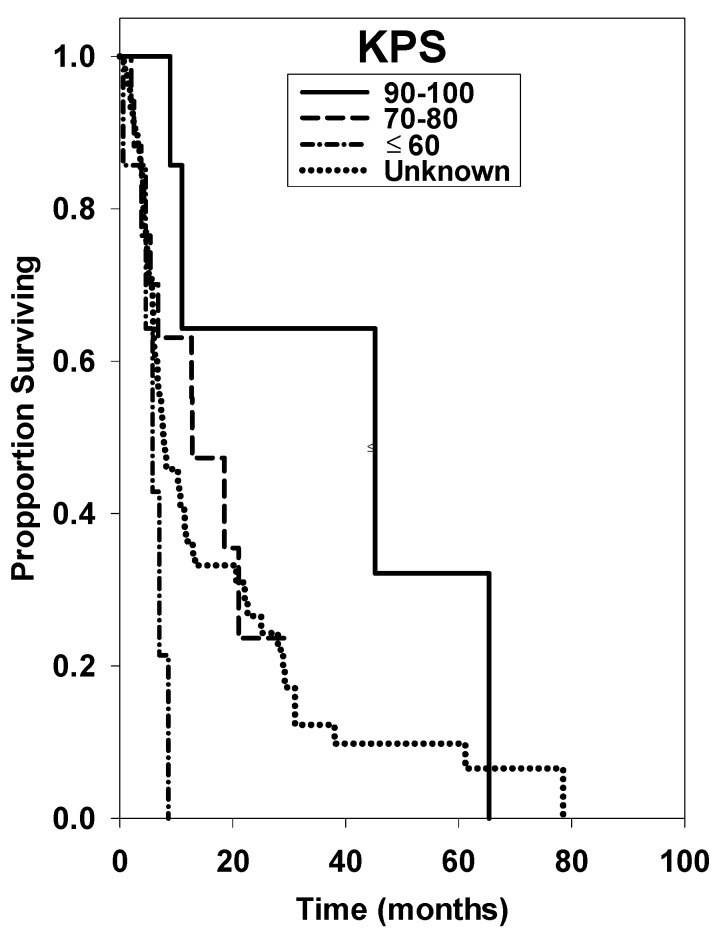
Overall survival curves of patients with Karnofsky Performance Score (KPS) of 90–100, 70–80, ≤60, or unknown at time of initial Gamma Knife radiosurgery (GKRS) treatment.

**Figure 3:**
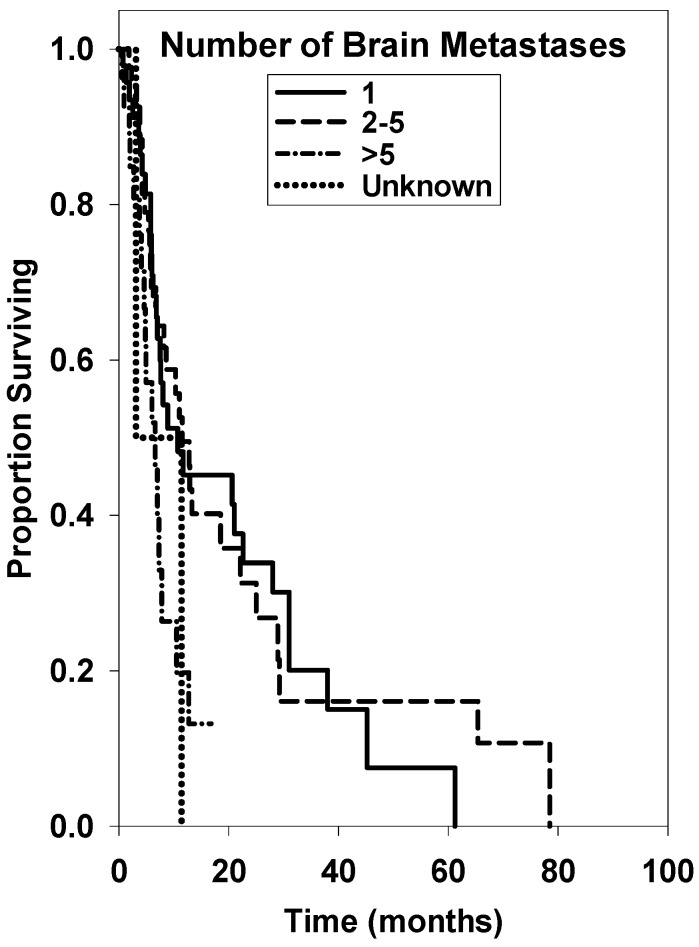
Overall survival curves of patients with 1, 2–5, >5, or unknown number of brain metastases on first diagnosis.

Kaplan-Meier survival curves for the several treatment groups can be found in Figure 1, Figure 2 and Figure 3. Figure 1 shows that a statistical difference did not exist for patients greater than age 65 compared with patients < age 65 (*p* = 0.784). Figure 2 displays survival curves based on KPS, with a significantly shorter survival time for KPS ≤ 60 (*p* ≤ 0.001). Figure 3 displays survival curves based on number of brain metastases, with >5 metastases being a significant negative prognostic factor (*p* = 0.024). Univariate median survival confidence interval and hazard ratio confidence intervals are included in Table 2. For each category a reference group was selected against which the other groups’ hazard ratios were tested.

The multivariate analysis hazard ratio estimates and confidence intervals are included in Table 3. The multivariate analysis utilized the same reference groups as the univariate analyses against which the other groups’ hazard ratios were tested. The multivariate analysis indicated patients with KPS = 70–80 and KPS ≤ 60 had survival experience that was significantly worse than the reference group (KPS = 90–100, *p* ≤ 0.001). In addition, patients with a number brain metastases >5 were found to have significantly decreased survival compared to the reference group (number brain metastases = 1, *p* ≤ 0.001). Receiving prior WBRT was not associated with a significantly longer survival time (*p* = 0.161). Absolute survival rates at 0.5, 1, 2, and 5 years are found in Table 4, displaying the poor 5 year survival rate in this cohort (8.1% at 5 years).

## 3. Discussion

Given the bleak prognosis for patients with intracranial melanoma or renal cell carcinoma, it is essential to identify prognostic factors to plan optimal treatments that can extend survival and maximize comfort. While SRS cannot be considered a curative therapy, it is an effective palliative treatment for brain metastases and can lead to improved survival in certain patients demonstrates excellent local control of intracranial disease. The goal of our study was to establish a relationship between pre-treatment clinical factors and overall survival in patients treated with SRS suffering from these radioresistant lesions.

The past gold-standard treatment for non-resectable brain metastases has been WBRT, but given the efficacy of SRS, it has been less used for the initial treatment of these radio-resistant lesions. Treatment with whole brain radiation has shown to increase rate of neurocognitive decline—a process often attributable to hippocampal damage and frequently impacting patients with longer prognoses [24,25]. Several studies found that patients undergoing the combination of SRS and WBRT experienced significantly higher rates of cognitive decline than patients only undergoing SRS [25,26]. The 2015 NCCTG N0574 study by Brown *et al.* [25] found that 91.7% of patients treated with both modalities experienced significant neurocognitive decline, compared to 63.5% of patients treated with SRS alone. The same study also found that while adding WBRT improved local tumor control, there was no difference in OS between patients treated with both modalities *versus* those treated only with SRS. Similarly, our study found no significant difference in median survival times on univariate or multivariate analysis between patients who had undergone previous WBRT and those who had not (*p* = 0.161, 0.669). Although the efficacy of SRS in extending OS in patients with all tumor histologies is still debated, its ability to provide local control and minimize neurological side effects is well established [11,22,25,27,28,29].

Multiple studies have found that SRS offers excellent local control of intracranial RCC and melanoma. Lwu *et al.* [28] determined local control rates of 75% and 91% at 12 months for melanoma and RCC, respectively, in patients treated with SRS alone. A study by Muacevic *et al.* [11] found a local control rate of 94% in intracranial RCC patients treated with SRS alone, and 78% of these patients died of progressive systemic cancer rather than neurologic compromise. Sheehan *et al.* [22] found that treating brain metastases from RCC with SRS in 69 patients provided a local control rate of 96%, while a 2015 study by Frakes *et al.* [29] of 28 melanoma patients treated with SRS determined 6 and 12 month local control rates of 91.3% and 82.2%, respectively.

Our results demonstrate an overall median survival rate of 12.7 and 8.2 months for RCC and melanoma, respectively. The same median survival in melanoma patients treated with SRS was determined by Mikoshiba *et al.* in 2013 [30]. Age at diagnosis was not a significant factor in overall prognosis, although patients <65 years of age had a slightly extended overall median survival time (*p* = 0.784). We also found that number of brain metastases prior to treatment and initial KPS are significant pre-treatment prognostic factors. The significance of our KPS scores is notably impacted by the small sample size of patients with documented scores at initial presentation. More thorough assessment and documentation will increase the validity of these findings through a larger sample size in future studies.

While the presence of multiple metastases has been negatively correlated to outcome before, the optimal treatment modality of these patients remains controversial [23]. Fokas *et al.* [31] determined addition of SRS to WBRT in RCC patients with 1–3 metastases resulted in superior intracerebral control, especially in RPA class 1 patients. That study suggested reserving treatments of only WBRT to patients with multiple metastases and very poor prognosis. Powell *et al.* [32] determined KPS to be a more significant prognostic indicator than number of metastases, suggesting SRS is effective in treating multiple, radioresistant brain metastases. Most recently, Frakes *et al.* [29] determined SRS to be useful in treating >5 lesions from metastatic melanoma. A prospective randomized study that examines the impact of adding WBRT in patients with multiple, radioresistant metastases is necessary to fully understand this important therapeutic role, as trials to date have not specifically addressed these histologic subgroups.

KPS has been found in multiple clinical studies to be a prognostic factor in overall survival [11,23,32,33]. A 2014 study by Dyer *et al.* [23] found KPS to be significant predictor of OS for patients with intracranial melanoma treated with SRS. Muacevic *et al.* [11] determined KPS > 70 was correlated to longer OS on univariate analysis in RCC patients treated with SRS. The Radiation Therapy Oncology Group’s (RTOG) has accepted KPS as one of its factors to categorize patients in different prognostic groups for a variety of cancers in its recursive partitioning analysis (RPA) [34]. To date, KPS is perhaps the most supported prognostic factor for patients with brain metastatic cancer, and it should always be considered in the prognosis and treatment of patients with RCC or melanoma undergoing SRS.

In our study, surgical resection was not associated with a longer survival time (*p* = 0.275). Contrarily, a 2014 study by Bennani *et al.* [21] found a statistically significant extension in OS in RCC patients with previous resection, although mostly in patients who presented with a single metastasis (*p* = 0.04) or superficial metastases (*p* = 0.02). Resection can be a necessary treatment modality, and is often accompanied with adjuvant radiation to the tumor bed in order to achieve ideal margins. Surgery is often not clinically recommended due to sensitivity of affected areas or the presence of multiple lesions, but is warranted in specific cases, especially when peritumoral edema or brain shift is present [35].

Previous studies have established additional prognostic factors. The study by Bennani *et al.* [21] found that absence of intracranial hypertension (*p* = 0.01), absence of acute metastasis (*p* = 0.03), and absence of extracranial metastasis (*p* = 0.049) were significant predictors of OS in patients with metastatic RCC treated with a variety of modalities. Dyer *et al.* [23] determined that extensive extracranial metastases (*p* = 0.001) were associated with OS on a multivariate analysis. A retrospective study by Mori *et al.* [6] found that absence of active systemic disease and presence of a solitary metastasis were associated with better OS in melanoma patients on multivariate analysis. A 2014 analysis of 135 melanoma patients undergoing SRS by Marcus *et al.* [36] found KPS (*p* = 0.02) and serum LDH levels (*p* = 0.01) to be prognostic factors significant for OS on multivariate analysis. Additionally, the parallels between our findings and established prognostic factors by the Diagnosis-Specific Graded Prognostic Assessment (DS-GPA) system should be noted [37]. These factors must be carefully considered when developing a treatment plan for each patient.

Systemic metastases from both RCC and melanoma have become targets for immunotherapeutic treatments as well. Ipilimumab, a monoclonal antibody that upregulates immune responses by targeting CTLA-4 (cytotoxic T-lymphocyte-associated protein 4), has been shown to prolong survival times in both previously treated and untreated metastatic melanoma patients when used in combination with other therapies [38,39]. The landmark study by Hodi *et al.* [38] demonstrated extended survival of more than 10 months in patients treated with ipilimumab (*p* ≤ 0.003), but few of these patients had brain metastases and survival date regarding this subset was not reported. Another study by Robert *et al.* [39] demonstrated similar results in patients without brain metastases. To examine the impact of adding ipilimumab to treatment of patients with intracranial melanoma and previous SRS treatment, Knisely *et al.* [40] analyzed 77 patients with intracranial melanoma treated with SRS. The 27 (35%) of patients receiving ipilimumab experienced a median survival of 21.3 months (*p* = 0.03), compared to a median survival of 4.9 months in patients who did not receive the therapy. Comparable results were seen in a recent study conducted by Scoenfeld *et al.* [41], in which intracranial melanoma patients treated with ipilimumab and SRS experienced a median survival of 14.4 months, with those who received SRS prior to ipilimumab experiencing a longer median survival compared to those who initiated ipilimumab prior to SRS (26 months compared with 6 months, *p* < 0.001). These studies offer convincing evidence of the benefits of adding ipilimumab to SRS in the treatment of particular patients with intracranial melanoma.

In addition, the use of other immunotherapies has been approved for treatment of advanced metastatic renal cell carcinoma. Inhibitors of mammalian target of rapamycin (mTOR) work by interfering with protein kinases that modulate protein synthesis and cell proliferation in cancers. The mTOR inhibitor everolimus has been shown to be an effective salvage therapy in patients with metastatic RCC, extending OS to 24 months (*p* = 0.047) [42]. Previous retrospective studies have determined similar benefits of everolimus, especially when compared to temsirolimus, another mTOR inhibitor previously consider a mainstay of therapy in these patients [43,44]. Nivolumab, a recently FDA-approved PD-1 inhibitor, has been shown to be more effective in prolonging overall survival in metastatic renal cell carcinoma patients than everolimus. (104). A study by Motzer *et al.* [45] of patients with advanced RCC demonstrated a 5.4-month survival benefit of nivolumab *versus* everolimus (*p* = 0.002). Such studies are supportive of nivolumab becoming a therapeutic mainstay in patients with advanced renal cell carcinoma, but further retrospective studies are needed to properly determine the survival benefit of PD-1 inhibitors in patients with intracranial renal cell carcinoma, and their therapeutic utility in combination with SRS.

## 4. Materials and Methods

For this analysis, we examined 122 patients with a diagnosis of brain metastasis and primary tumor histology of kidney cancer or melanoma. We included patients who were treated between December 2001 and July 2014. IRB approval for this study was obtained prior to initiation by the Spokane IRB (study number 1507). Individual patient consent was deemed not necessary because of the retrospective nature of the analysis. The patients were grouped by age at brain metastasis diagnosis (<65, 65+, Unknown), by whether or not they received WBRT, by whether or not they underwent resection, by KPS value (90–100, 70–80, ≤60, Unknown), by number of brain metastases (1, 2–5, >5, Unknown), and by primary tumor histology (RCC, melanoma). Patients underwent GKRS from a Leksell Gamma Knife^®^ Model C or Leksell Gamma Knife^®^ Perfexion.

Survival curves were estimated using the Kaplan-Meier method and used to compare age groups, KPS groups, primary tumor histology groups, brain metastases number groups, and treatment groups. Andersen 95% confidence intervals for median survival times of each group were determined. Confidence intervals for the hazard-ratio were calculated using the estimate of standard error (*se*):

(1) $$se=\sqrt{\overset{k}{\underset{i-1}{\sum }}\frac{1}{e_{ij}}}$$

where *e_i_* is the extent of exposure to risk of death for group *i* of *k* at the *j*th distinct observed time for group *i* of *k* (Armitage P, Berry G. Statistical Methods in Medical Research (3rd edition). Blackwell 1994.) Log-rank tests were used to determine if there is a statistical difference between the survival rates of the different groups. The Cox proportional hazard model was used in a multivariate analysis of the age groups, KPS groups, primary tumor histology groups, and the treatment groups. All statistical analyses utilized StatsDirect Version 2.8.0 (StatsDirect Ltd., Altrincham, UK) and SigmaPlot Version 11.0 (SYSTAT Software, Inc., San Jose, CA, USA).

## 5. Conclusions

RCC and melanoma commonly metastasize to the brain and can cause devastating neurological damage. Surgery is not always possible, and WBRT is of questionable use due to the radioresistant nature of these lesions. SRS has proven effective in treating these lesions in multiple clinical reports, demonstrating both high local control rates and extended overall survival while significantly decreasing neurocognitive damage. Our retrospective study determined high KPS and a lower number of intracranial metastases to be significant prognostic factors for improved overall survival in patients with intracranial RCC or melanoma treated with SRS.

## Figures and Tables

**Table 1:** Patient population baseline characteristics.

Treatment Group	Primary Tumor		
	RCC	Melanoma	Total
	(*n* = 28)	(*n* = 94)	(*n* = 122)
*Age at Diagnosis*			
<65	18	71	89
≥65	9	22	31
Unknown	1	1	2
KPS			
90–100	4	8	12
70–80	3	14	17
≤60	1	6	7
Unknown	20	66	86
*# Brain Mets*			
1	9	38	47
2–5	13	33	46
KPS > 5	5	22	27
Unknown	1	1	2
*Received WBRT*			
No	23	77	100
Yes	5	17	22
*Underwent Resection*			
No	23	68	91
Yes	5	25	30
Unknown	0	1	1

#—Number of brain mets; KPS—Karnofsky Performance Score.

**Table 2:** Univariate median survival estimates (months) and hazard ratios.

Treatment Group	Median Survival		Hazard Ratio		
	*n*	95% CI	Estimate	95% CI	*p* Value **
*Age at Diagnosis*					
<65 *	89	10.30 ± 3.49	reference		
≥65	31	8.60 ± 6.27	1.09	0.61–1.88	0.784
Unknown	2	1.30 ± unknown	7.99	0.18–63.46	0.133
*KPS*					
90–100 *	12	45.20 ± 43.52	reference		
70–80	17	12.80 ± 7.64	3.28	0.69–31.14	0.139
≤60	7	5.80 ± 2.46	7.90	1.18–52.97	<0.001
Unknown	86	7.80 ± 3.24	2.87	1.05–10.95	0.035
*# Brain Mets*					
1 *	47	10.70 ± 13.40	reference		
2–5	46	11.50 ± 3.36	0.95	0.55–1.67	0.895
>5	27	6.60 ± 2.45	2.24	1.09–4.51	0.024
Unknown	2	3.10 ± unknown	2.42	0.27–10.09	0.219
*WBRT Received*					
No *	100	10.70 ± 3.34	reference		
Yes	22	7.00 ± 1.89	3.10	0.80–2.57	0.161
*Resection Undergone*					
No *	91	8.60 ± 3.16	reference		
Yes	30	11.40 ± 18.13	0.74	0.42–1.25	0.275
Unknown	1				
*Primary Tumor*					
RCC *	28	12.70 ± 2.63	reference		
Melanoma	94	8.20 ± 3.06	1.21	0.70–2.19	0.519

* Reference group against which other groups’ survival experience are compared; ** *p* value for log-rank testing the null hypothesis that the groups’ survival experience is same as reference group.

**Table 3:** Multivariate hazard ratios, confidence intervals, and *p* values.

Treatment Group	*n*	Hazard Ratio		
		Estimate	95% CI	*p* Value **
*Age at Diagnosis*				
<65 *	89	reference		
≥65	31	1.03	0.60–1.77	0.906
Unknown	2	57.16	5.41–603.98	<0.001
*KPS*				
90–100 *	12	reference		
70–80	17	1.67	1.20–2.34	<0.001
≤60	7	5.56	5.17–5.97	<0.001
Unknown	86	2.43	0.85–6.90	0.100
*# Brain Mets*				
1 *	47	reference		
2–5	46	1.04	0.61–1.79	0.880
>5	27	2.30	2.09–2.53	<0.001
Unknown	2	2.98	1.16–7.68	0.020
*WBRT Received*				
No *	100	reference		
Yes	22	1.14	0.63–2.06	0.669
*Resection Undergone*				
No *	91	reference		
Yes	30	0.72	0.41–1.28	0.267
Unknown	1	-	-	-
*Primary Tumor*				
Kidney *	28	reference		
Melanoma	94	1.21	0.70-2.17	0.496

* Reference group against which other groups’ survival experience are compared; ** *p* value for test if groups’ survival experience is same as reference group.

**Table 4:** Absolute survival rates at 0.5, 1, 2, and 5 years.

Year	Survival Rate	95% CI
0.5	65.7	55.9–73.9
1	40.0	30.0–49.7
2	26.0	16.7–36.3
5	8.1	2.4–18.1

## Abbreviations

The following abbreviations are used in this manuscript:

RCC: Renal cell carcinoma
SRS: Stereotactic radiosurgery
OS: Overall survival
WBRT: Whole brain radiation therapy
KPS: Karnofsky Performance Status
